# A hydrodynamic antenna: novel lateral line system in the tail of myliobatid stingrays

**DOI:** 10.1098/rspb.2024.2192

**Published:** 2025-01-22

**Authors:** Júlia Chaumel, George V. Lauder

**Affiliations:** ^1^ Department of Organismic and Evolutionary Biology, Harvard University, 26 Oxford Street, Cambridge, MA 02138, USA

**Keywords:** stingray, Myliobatidae, tail, vertebral column, flow sensing, neuromast

## Abstract

Eagle rays, cownose rays and manta rays (order Myliobatiformes) have a slender tail that can be longer than the animal’s body length, but its function and structure are unknown. Using histology, immunohistochemistry and three-dimensional imaging with micro-computed tomography scans, we describe the anatomy and function of the tail in *Rhinoptera bonasus*, the cownose ray. The tail is an extension of the vertebral column with unique morphological specializations. Along the tail behind the barb, vertebral centra are absent and neural and haemal arches fuse to form a solid mineralized structure that we describe for the first time and term *caudal synarcual*, which imparts passive stiffness to the tail, reducing bending. Two lateral line canals connected to an extensive tubule network extend along both sides of the tail. Tubules branch from the lateral line canal toward the dorsal and ventral tail surfaces, opening to the surrounding water via pores. A continuous neuromast is located within each lateral line canal, maintaining an uninterrupted structure along the entire tail. The complex lateral line mechanosensory system in the tail of *R. bonasus* supports the hypothesis that the tail functions like a hydrodynamic sensory antenna and may play an important role in their behavioural and functional ecology.

## 1. Introduction

Batoids (rays and skates) constitute the largest group of cartilaginous fishes, which also includes sharks and chimaeras [1]. Batoids are characterized by a dorsoventrally flattened body and are distributed worldwide, inhabiting a wide range of depths (up to 3000 m), primarily in benthic and epibenthic ecosystems [1,2]. The only batoids with a pelagic lifestyle comprise four families within the order Myliobatiformes: Myliobatidae (eagle rays), Aetobatidae (pelagic eagle rays), Rhinopteridae (cownose rays) and Mobulidae (devil and manta rays) [1], which are referred to here as myliobatids. Their adaptation to a pelagic lifestyle is reflected in their distinctive body morphology, locomotion style, and how they interact with and sense their environment [3,4]. Myliobatids have a triangular body shape with enlarged pectoral fins that oscillate vertically to move the animal forward, allowing them to swim effectively at different speeds and migrate long distances [3,5]. They also possess a well-developed mechanosensory lateral line system (LLS) on the head and body, as well as an electrosensory system that is predominantly located on the ventral body surface in those species that detect prey buried in the seabed [6–8]. However, as pelagic species, it remains unclear how myliobatids use these senses to detect stimuli, such as predators approaching from behind or localizing conspecifics during the formation of large schools, characteristic of many species within these families [9].

**Figure 1.**
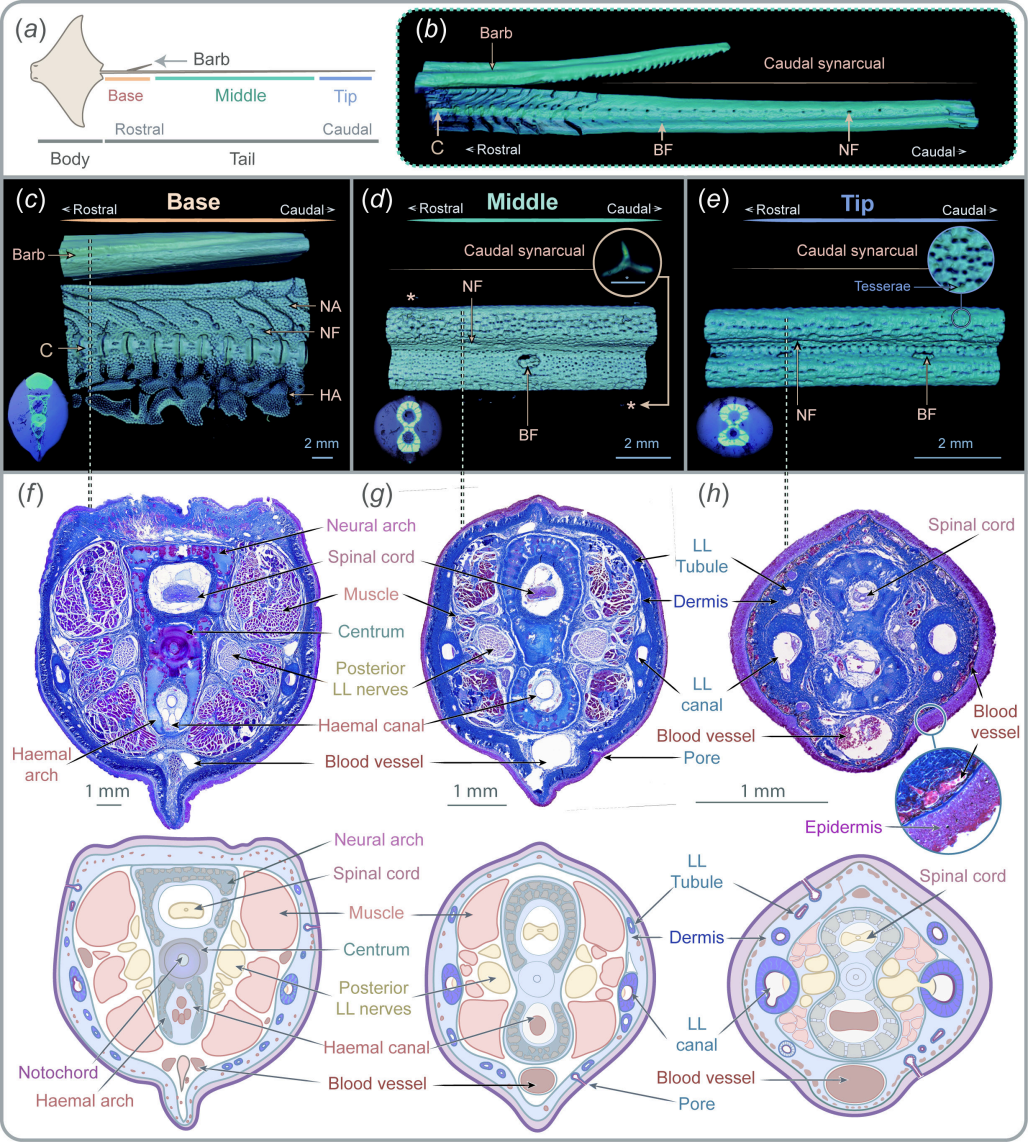
Tail anatomy**.** (***a***) Tail division into base, middle and tip zones. (*b*) Micro-computed tomography (µCT) scan showing the transition from segmented vertebrae to the caudal synarcual, with blood and nerve foramina following similar locations as in anterior vertebrae. (*c*) µCT scan of the tail base, characterized by separated vertebrae units with neural arches, haemal arches and by a well-defined centrum. Nerves divide from the spinal cord and traverse nerve foramina, while blood vessels divide from the haemal arch and transverse blood foramina. (*d*) µCT scan of the middle zone with caudal synarcual covered by tesserae and lacking centra. This is the only zone with denticles. (*e*) µCT scan of the tip zone showing the continuity of the caudal synarcual until the tail end. (*f*–*h*) Cross-sections of each tail zone stained with Mallory trichrome with an illustrative guide to the key structures below. Note variation in vertebral morphology and the consistent presence of the spinal cord and the haemal canal. Muscles (red) are predominant at the base and are gradually reduced in the middle and tip zones. In all zones, posterior lateral line nerves (lilac colour) run parallel to the vertebral column. The lateral line canals (both at each side of the tail) and the tubules are embedded in the dermis, while surface pores are located in the epidermis. BF, blood foramina; C, centrum; *, denticles; HA, haemal arch; LL, lateral line; NA, neural arch; NF, nerve foramina.

In addition to their specialized body shape, myliobatids are characterized by having a long and slender tail (often referred to as the whip-tail), which extends caudally from the body and supports a dorsal barb located at the tail base (figure 1*a*; [1]). The whip-like tail is an exclusive feature of myliobatiforms and is absent in other batoids or chondrichthyans [1]. Unlike most bony fishes and sharks [10,11], myliobatids do not use their tails as a propulsive structure. Although low amplitude movement of the myliobatid tail occurs during steady locomotion and manoeuvering, the tail generally maintains a rostro-caudal orientation behind the body during swimming [3]. This positioning suggests that the tail may have a sensory role, potentially detecting approaching predators or providing information on water flow and body position. However, no anatomical or functional studies on myliobatid’s tail are currently available to corroborate this proposed sensory function.

The overall aim of this study is to define the anatomy of the whip-tail of *Rhinoptera bonasus* (cownose ray)*,* including a detailed characterization of its LLS and vertebral column, which may provide insights into a more comprehensive understanding of the biological function of the myliobatid tail. Our approach involves a combination of morphometric, gross anatomical, three-dimensional imaging and histological analysis using tails of cownose rays, a species with a well-documented ecology that inhabits diverse ecological niches [12,13]. Specifically, we describe the morphological features of the tail and how they vary along its length, characterize the remarkable three-dimensional structure of the LLS (including the lateral line canal (LLC), tubules, pores, the continuous neuromast and nerves) that extends along the entire tail and propose a potential function as a hydrodynamic filtering system. Understanding the tail anatomy in cownose rays lays the groundwork for future comparative studies of tail anatomy in other ray species with diverse ecologies, as well as behavioural and biomechanical analyses of tail function during locomotion.

## 2. Methods

### (a) Sample collection

A total of eight specimens of *R. bonasus,* including males and females of different sizes, were used in this study (electronic supplementary material, table S1). Animal sizes were defined by disc width (DW), described as the distance between the tips of the pectoral fins. For gross morphometrics and comparative analysis, six individuals from the ichthyology collection at the Museum of Comparative Zoology of Harvard University were used (electronic supplementary material, table S1). These samples are stored in 70% ethanol. For fine-scale histology, two fresh specimens were obtained from Ripley’s Aquarium (DW = 81 cm, male), where tails were collected immediately after the natural death of the animal, and GULFSPAN survey (Gulf of Mexico Shark Pupping and Nursery survey) [14] in Florida (DW = 50 cm, male), where the animal was euthanized following the ethical procedure under U.S. Fish and Wildlife Permit #SAL-1918-SRP (electronic supplementary material, table S1). Tails from fresh specimens were cut into small pieces (around 1 cm length for histology and 2–4 cm length for X-ray micro-computed tomography (µCT)) immediately after the animal’s death and fixed in 4% paraformaldehyde (PFA; Santa Cruz Biotechnology Inc., Dallas, USA) in phosphate-buffered saline (PBS) during 6 h at room temperature. Tails were divided into three zones: the base (from the end of the pelvic fins to the posterior end of the barb), the middle zone (from the end of the barb until the tip zone) and the tip zone (last 15% of the tail, approximately 5 cm in length; figure 1*a*). Based on the tail anatomy, each zone varied in length, resulting in different sample counts. For histology and immunofluorescence, 10 samples were analysed per animal (three base, four middle and three tip), while µCT scans included five samples per animal (two base, two middle and one tip).

### (b) X-ray micro-computed tomography

High-resolution µCT scans were conducted to image in three-dimensions the tissues forming the tail, enabling fine-scale morphological analyses. Samples fixed in 4% PFA were washed in PBS and transferred into increasing ethanol concentrations (10%, 30% and 50%) up to 70% ethanol. High-resolution µCT images were obtained using a Bruker SkyScan 1273 (70 kV and 114 µA). Image pixel size ranged between 4.9 and 13 µm. Samples were post-stained in 0.3% phosphotungstic acid (PTA; AMRESCO Inc., Solon, USA) following [15] for over a month, changing the PTA solution every 2 weeks to assure complete staining. After staining was complete, PTA-stained samples were transferred to 70% ethanol and rescanned. Images were reconstructed with Bruker Nrecon software and visualized, segmented and analysed with Amira-Avizo visualization software v. 2023.11 (Thermo Fisher Scientific Inc., Waltham, USA).

### (c) Histology and Immunofluorescence

After fixation in 4% PFA, samples were washed in PBS, decalcified using Epredia Decalfying Solution (Thermo Fisher Scientific Inc., Waltham, USA) for 48 h, and dehydrated in ethanol solutions of increasing concentrations, ending with immersion in 100% ethanol. Samples were transferred to 100% xylene, embedded in paraffin, cut into 5−7 µm thick sections with a microtome, mounted in VistaVision™ HistoBond^®^ Premium Adhesion Slides (VWR International, Radnor, USA) and left to dry on a warming tray at 37°C overnight.

Histology was used to identify the different tissues and their spatial organization in the tail. Sections were stained with haematoxylin–eosin combined with Orange G or with Mallory trichrome (protocols customized from [16]). Haematoxylin (purple) is a basic stain that binds to acidic structures (e.g. nuclei), while eosin (pink) and Orange G (orange) are acidic stains with affinity for basic structures (e.g. cartilage matrix). The combination of both acidic stains helps to enhance different connective tissues by providing additional contrast. Mallory trichrome was used to identify muscle (red to orange), nervous tissue (lilac), collagen (dark blue), dense cellular tissue (pink to purple), mucus and connective tissue (blue), myelin and red blood cells (pink to red), and nuclei (red). Histology-stained samples were imaged using a Keyence digital microscope VHX-600 (Keyence Corporation, Osaka, Japan) and a Leica light microscope (Leica Microsystems GmbH, Wetzlar, Germany). Images were analysed using Fiji 2.0.0.

Immunofluorescence staining was used to identify nervous tissue within the tail by staining the sections with β-III-tubulin antibody. For antigen retrieval, samples were incubated in 10 mM sodium citrate buffer with 0.05% Tween 20 (pH 6) for 6 h at 60°C. Samples were permeabilized in PBS with 0.025% Triton X-100 and blocked with 5% bovine serum albumin (BSA) in PBS (Sigma-Aldrich, St. Louis, USA) for 2 h at room temperature. The primary antibody β-III-tubulin (1:500; Cat# ab18207, Abcam, Cambridge, UK) was used as a neuronal marker due to its previously demonstrated reactivity with catshark tissues [17]. Samples were incubated with the primary antibody in 1% BSA in PBS overnight and then incubated for 1 h at room temperature with the secondary antibody goat anti-mouse IgG H&L Alexa Fluor 488 (1:500; Cat# ab150113, Abcam, Cambridge, UK) diluted in 1% BSA and then rinsed in PBS. Some tissue structures displayed an autofluorescence signal at 488 nm. To avoid interference with the antibody signal, samples were stained with 0.1% Sudan Black B (Sigma-Aldrich, St. Louis, USA) following protocols established by [18]. Samples were washed in PBS to remove excess stain and mounted with SlowFade™ Diamond antifade mountant with 4′,6-diamidino-2-phenylindole (DAPI; Thermo Fisher Scientific Inc., Waltham, USA). This mountant with DAPI added additional autofluorescence signal when the samples were excited with a 405 nm laser, especially in the dermis. However, this did not affect the visualization of the structures of interest. Images of sections stained with immunofluorescence were acquired using a Zeiss LSM 980 confocal microscope (Carl Zeiss GmbH, Oberkochen, Germany) and a Leica DM R fluorescence microscope (Leica Microsystems GmbH, Wetzlar, Germany). Images were analysed using Fiji 2.0.0.

Negative controls were performed (electronic supplementary material, figure 2). The autofluorescence signal was stronger when the samples were excited with a 405 nm laser and became more pronounced by increasing image brightness. This signal was used to obtain greyscale images that showed the general tissue anatomy and served as reference for the position of the structures of interest in the section, following [19].

### (d) Measurements and analysis

**Figure 2.**
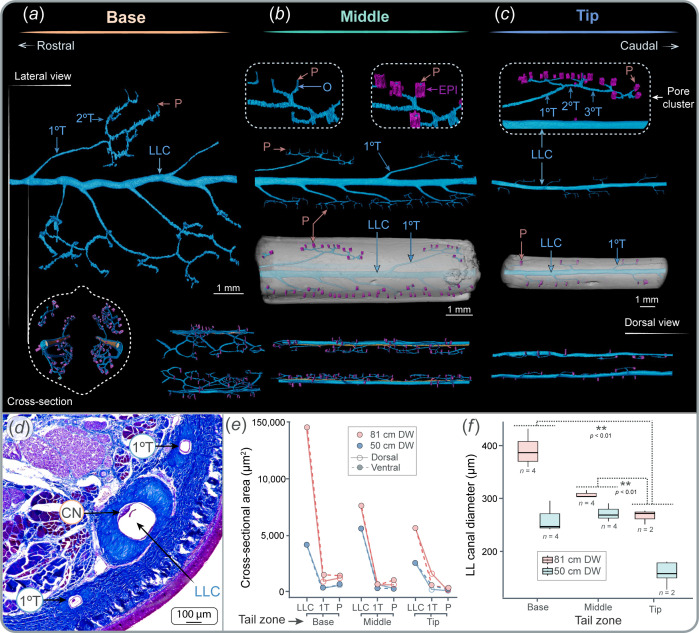
Lateral line morphology along the tail. (*a*–*c*) µCT reconstructions of the lateral line canal (LLC, blue), tubules (blue) and pores (pink) in the three tail zones. The first tubules to divide from the LLC are the primary tubules. Primary tubules continue dividing, forming secondary tubules which further divide until they reach the epidermis and open via pores. Pores connected to a common primary tubule form a *pore cluster*. Cross-section (dashed lines represent the tail surface) and dorsal view show the position of both LLCs on each side of the tail. (*d*) Mallory trichrome staining showing the LLC, primary tubules and the canal neuromast. (*e*) Variation in the cross-sectional area of the LLC, tubules and the sum of the cross-sectional area of pores from the same pore cluster across tail zones, individuals and dorsal and ventral positions. (*f*) Variation in LLC diameter across tail zones and individuals. A double asterisk indicates statistical difference and *n* indicates sample size. Branch, B; epithelium, EPI; lateral line canal, LLC; pore, P; primary tubule, 1°T; secondary tubule, 2°T; tertiary tubule, 3°T; opening, O.

Morphometrics of specimens’ body (total length, body length and DW) and tails (tail length and diameter) were measured *in situ* (electronic supplementary material, table S2). PTA-stained samples were imaged using µCT and segmented with Amira Avizo, creating three-dimensional models of the LLC, tubules and pores (measured morphological parameters are summarized in electronic supplementary material, table S2; figure 2). The branching pattern of tubules was characterized using Amira Avizo. The diameters and lengths of the LLC, tubules and pores from both sides of the tail were measured using Amira Avizo and Meshlab. Diameters of LLC were calculated as the mean of multiple measurements taken along the LLC for each side, zone and animal. The sum of the cross-sectional area of pores connected to the same primary tubules (termed *pore cluster* in §3) was compared with the cross-sectional area of the LLC and primary tubules. Pore density was calculated as the number of pores per cm of tail for each tail zone in four specimens (electronic supplementary material, table S1). Statistical analyses were conducted to identify correlations between DW and tail length, assess variations in LLC diameter and pore size across tail zones and animal size, and identify differences in pore density across tail zones and animal size. Analyses were performed using linear models, ANOVA and Tuckey post hoc tests using R 2023.12.0+369.

## 3. Results

### (a) Tail morphometrics

*Rhinoptera bonasus* is characterized by having a long and slender tail with a cone-like tapering geometry (i.e. tail diameter decreases from the base toward the tip). Larger animals have larger and wider tails; however, the tail does not scale proportionally with the animal’s size (measured as DW; electronic supplementary material, figure S1A). In smaller animals, tails are 1.7 times larger than the DW (1:1.7 ratio), while in larger animals, the tail has a similar length to the DW (1:1 ratio; electronic supplementary material, figure S1B).

### (b) Tail anatomy

Micro-CT data show that the tail contains an extension of the vertebral column that elongates from the relatively thick base toward the thinner tip (figure 1). However, the morphology of the vertebral column varies across different tail zones. At the base (from the tail attachment location to the posterior end of the barb; figure 1*a*–*c*), the vertebral column is composed of a series of repeated vertebral units characterized by a centrum (an areolar calcified hourglass-shaped structure) that surrounds the notochord, by dorsal neural arches that encase the spinal cord and by ventral haemal arches that protect the caudal artery and vein (figure 1*b*,*c*). Both neural and haemal arches are covered by tesserae (mineralized cartilage), which is perforated by foramina that allow nerves and blood vessels to connect the caudal artery, veins and spinal cord to the surrounding tissues. After the posterior end of the barb and proceeding distally to the middle and tip zones, the centrum is absent, and the neural and haemal arches fuse, forming a solid element that extends to the tip (figure 1*b*,*d*,*e*). This solid structure is also covered by tesserae and contains the spinal cord (dorsally) and artery and caudal vein (ventrally), both connected to the surrounding tissues through nerve and blood foramina. Posterior lateral line nerves (PLLNs) run parallel to the vertebral column in a caudal direction, connecting the neuromasts to the brain (figure 1*f*–*h*, electronic supplementary material, figure S2). The vertebral column and PLLN are both surrounded by muscles; however, these muscular bundles are predominantly located at the base of the tail and almost absent at the tip (figure 1*f*–*h*).

**Figure 3.**
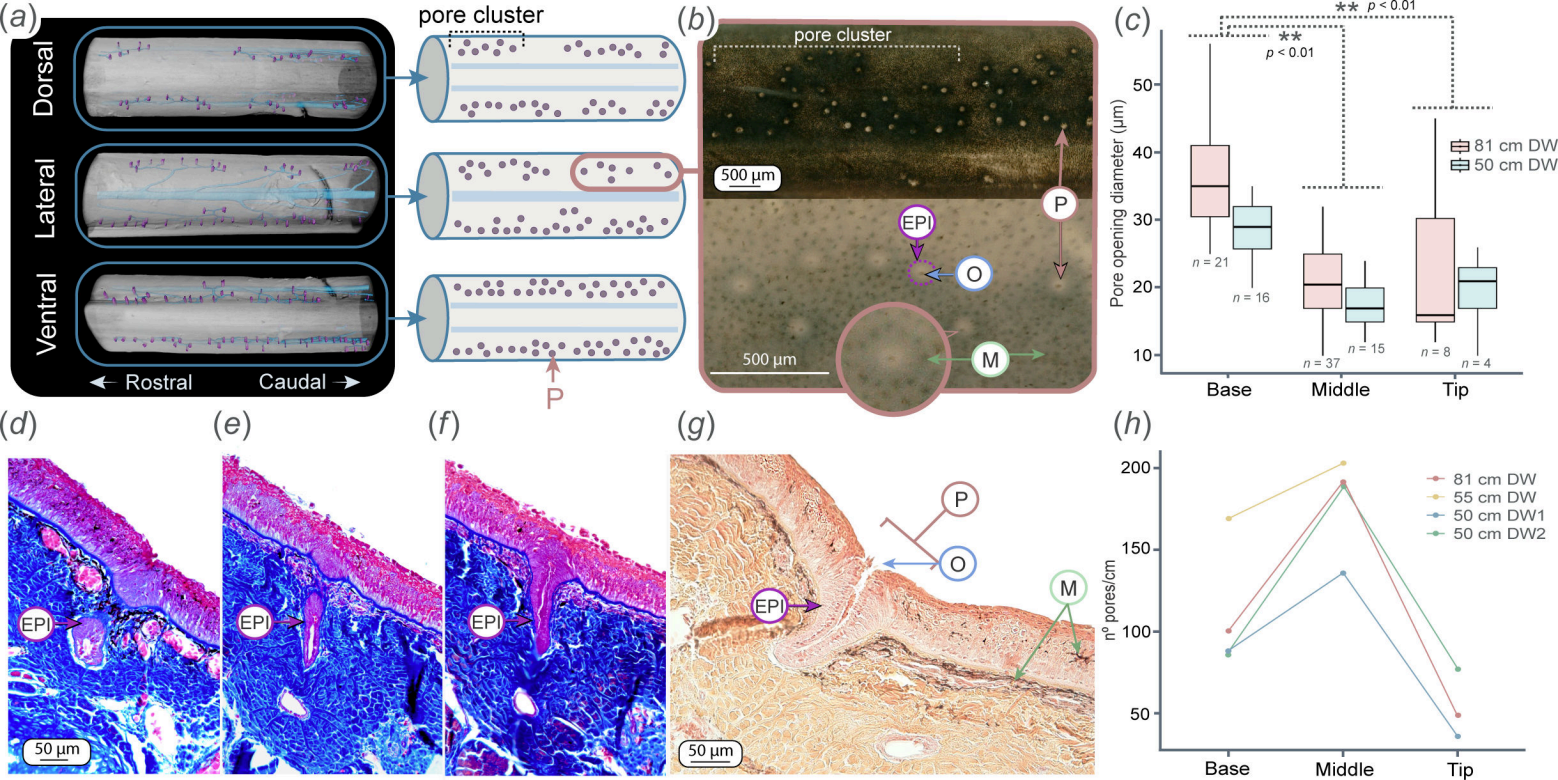
Pore distribution and morphology along the tail. (***a***) µCT reconstructions and diagrams showing pore distribution on the dorsal, lateral and ventral sides of the tail. Pore clusters consist of a group of pores that belong to the same primary tubule. Note the lack of pores on the lateral sides of the tail. (*b*) Light microscopy images showing pore distribution at the tail surface. Pores consist of an opening surrounded by an epithelial rim of columnar cells. Melanophores give dark coloration to the skin. (*c*) Variations in pore opening diameters across tail zones for two individuals. Asterisks indicate significant differences and *n* indicates sample size. (*d, e, f*) Mallory trichrome-stained sections showing a pore opening in sequential sections. (*g*) Haematoxylin-eosin and Orange G histology sections showing a pore opening, with the lumen surrounded by the epithelium. Note the melanophores located in the epidermis and dermis. (*h*) Pore density (n°pores/cm) varies significantly across tail zones and among animals of different sizes. The tip for the 55 cm DW individual was missing. Epithelium, EPI; melanophores, M; pore, P; pore opening, O.

Skeletal elements and muscles are further surrounded by a thick dermis (figure 1*f*–*h*). The dermis is further surrounded by an epidermis, formed of several layers of columnar cells (figure 1*h*). Embedded within the epidermis, and exclusively in the middle zone, the tail is covered by small (approximately 100 µm) spiny denticles (figure 1*d*). Dorsally, melanophores were observed in the dermis, both in the dermis and epidermis (figure 3*g*).

### (c) The mechanosensory lateral line

The tail contains an LLC connected to an extensive network of tubules that extends, embedded in the dermis, along both lateral sides of the entire tail (figure 2, electronic supplementary material, video S1, figure S3). The tubule network has a tree-like topology, with primary tubules extending from the LLC in both dorsal and ventral directions (figure 2*a*–*c*, electronic supplementary material, figure S3). Primary tubules are the initial and largest tubules branching from the LLC. Primary tubules further divide into secondary tubules, which then divide again into tertiary tubules, which continue dividing until reaching a maximum of eight divisions (figure 2*a*–*c*). Tubules eventually open to the environment via pores (figures 2 and 3). On the skin surface, pores are organized into groups of 15–20 pores, with each group deriving from a single primary tubule (figures 2*a*–*c* and 3*a*). We refer to these groups of pores as ‘pore clusters’ (figures 2*a*–*c* and 3*a*–*b*). With each division, the diameter of the tubules decreases, with the LLC having the largest diameter and the pores the smallest (figures 2*e*–*f* and 3*c*, electronic supplementary material, table S3). A reduction in diameter was also observed when comparing the cross-sectional area of the LLC and primary tubules to the cumulative cross-sectional area within the same pore cluster, although primary tubules and pore clusters maintain similar values (figure 2*e*).

The LLC, tubules and pores vary in morphology, size and branching order across base, middle and tip tail zones. The diameter of the LLC decreases significantly from the base to the tip (*F* = 34.54, d.f. = 2, *p* < 0.01; figure 2*f*, electronic supplementary material, table S3) and is positively correlated with the size of the animal, where larger individuals have larger LLC diameters (*F* = 51.89, d.f. = 1, *p* < 0.01; figure 2*f*). The number of tubule divisions from the LLC to the pores also varies across tail zones. The middle zone, which comprises the longest proportion of the tail, has the greatest number of branches and the highest pore density (number of pores per cm of tail; figure 3*h*, electronic supplementary material, table S3). In contrast, the tip has the lowest number of divisions, particularly in smaller individuals, where the tubule network has only two divisions before reaching the surface pores. This correlation and division pattern were similar for individuals of different sizes (figure 3*h*).

Pores are distinguished by the pore opening (defined here as the lumen) enveloped by a thick rim of epithelial cells (figure 1*a*,*b*). Pores are distributed dorsally and ventrally following a linear pattern but are not present at the lateral surfaces of the tail (figure 3*a*). Pore diameter (measured only by considering the pore opening, figure 3*b*) varied across tail zones, having significantly larger diameters at the base, with no differences observed between the middle and the tip (figure 3*c*, *F =* 63.10, d.f. = 2, *p <* 0.01; electronic supplementary material, table S3). Similar to the LLC, pore diameter also increased with the size of the animal (*F =* 18.05, d.f. = 1, *p <* 0.01; figure 3*c*; electronic supplementary material, table S3). Both the LLC and tubules are surrounded by a thick layer of collagenous connective tissue, which becomes thinner as the canals and tubules decrease in diameter (figures 1*f*–h and 2*d*).

### (d) Neuromasts

**Figure 4.**
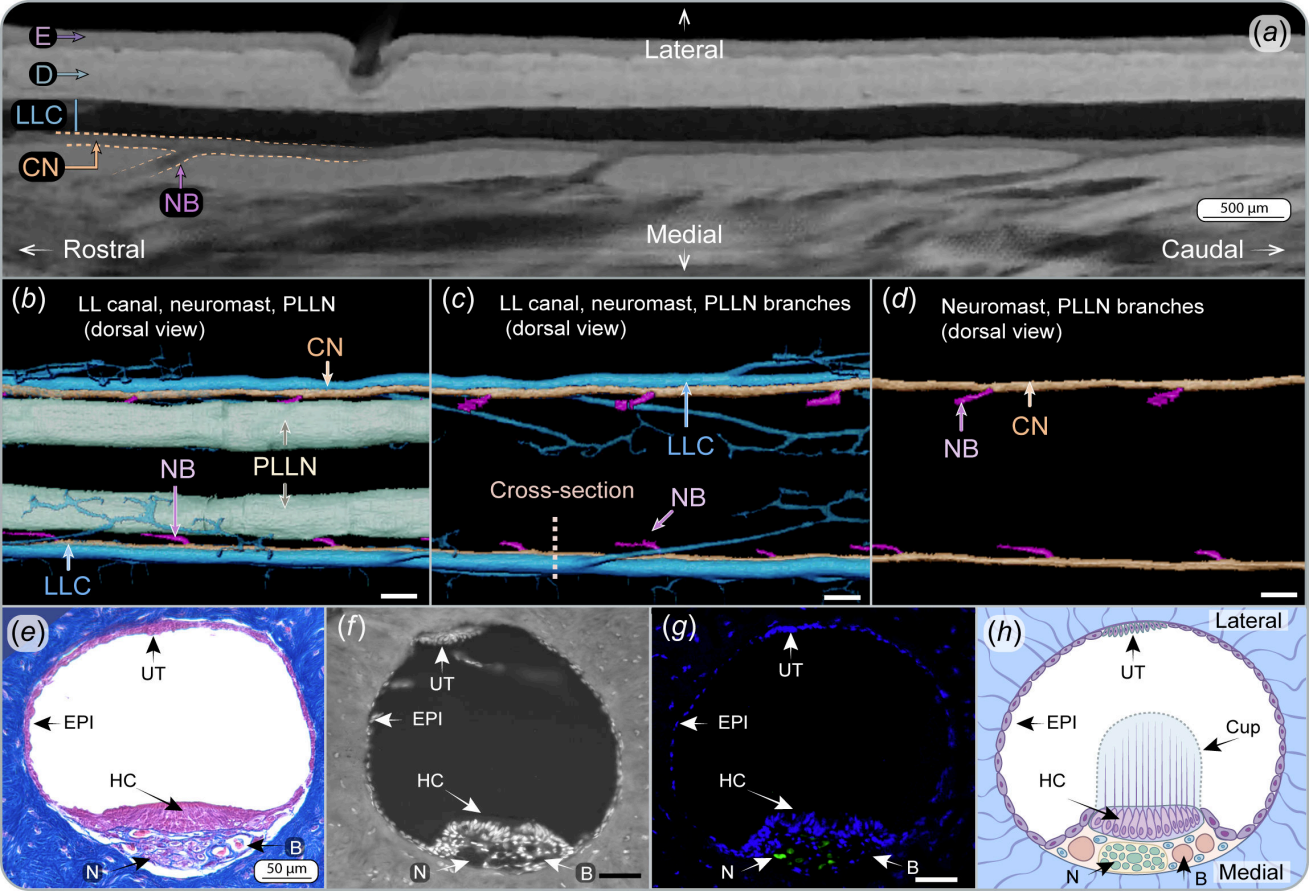
Anatomy of the continuous neuromast. (***a***) µCT-scan of a PTA-stained tail showing the continuous neuromast enclosed in the lateral line canal (LLC), with nerve branches periodically innervating the neuromast. Dermis and epidermis are visible. (*b–d*) Three-dimensional reconstruction from µCT scans showing the LLCs and tubules (blue), the continuous neuromast (orange), nerve branches (pink) and posterior lateral line nerves (PLLNs, green) that connect the neuromast to the brain. Nerve branches are positioned between two primary tubules, connecting the neuromast with the PLLNs. Dashed line in (*c*) indicates cross-sectional orientation of (*e–h*). (*e*) Mallory trichrome-stained section of the LLC and the continuous neuromast. (*f*) Autofluorescence image obtained with confocal microscopy showing the cellular nuclei (bright dots) and the surrounding dermis. (*g*) Fluorescence image showing the cell nuclei stained with DAPI (blue) and neural tissue stained with ß-III-tubulin antibody (green). (*h*) Interpretation of the structural organization of the LLC and neuromast, with the cupula based on the authors’ interpretation. Blood vessel, B; continuous neuromast, CN; cupula, Cup; dermis, D; epidermis, E; epithelium, EPI; hair cells, HC; lateral line canal, LLC; nerve, N; nerve branch, NB; primary tubule 1°T; posterior lateral line nerve, PLLN; unidentified tissue, UT. Scale bars in *f*–*g* = 50 µm. Scale bars in *b*–*d* = 500 µm.

Neuromasts were exclusively located in the LLC and were not observed in any of the tubules that lead to the pores. A combination of µCT scans, histology and immunofluorescence showed that the canal neuromast consists of a continuous structure along the entire tail, with no interruptions detected in any of our results (figure 4, electronic supplementary material, video S2). The continuity of the neuromast was particularly evident in PTA-stained and µCT-scanned samples, which allowed imaging of large tail sections (approximately 4 cm) (Figure 4*a*). The PLLN runs longitudinally along the tail beneath the canal (figure 4*b*). Nerve branches innervate the neuromast at regular intervals, always at constant distances between the division of two primary tubules (figure 4*a*–*d*, electronic supplementary material, video S2).

**Figure 5.**
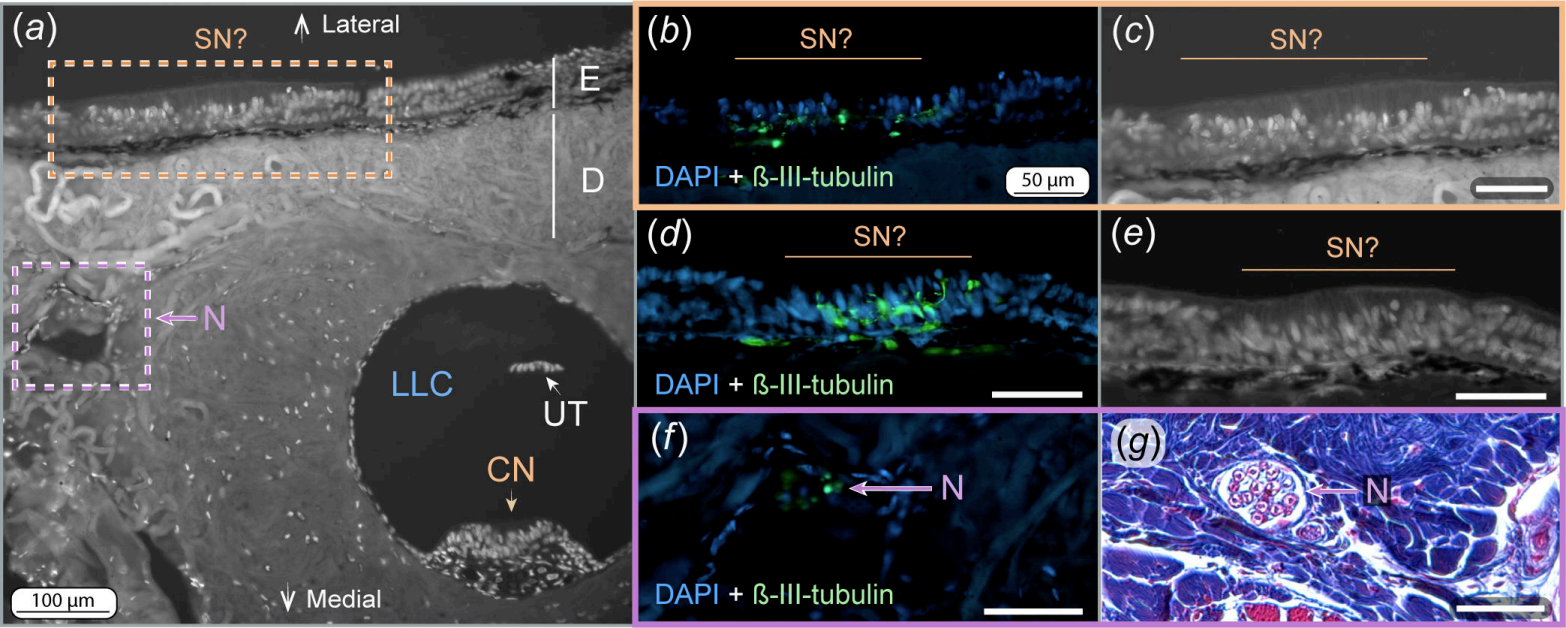
Putative superficial neuromasts. (*a*) Autofluorescence image showing the dermis with the LLC containing the continuous neuromast, an ascending nerve and the epidermis containing the sensory structures identified as potential superficial neuromasts. The unidentified tissue is separated from the canal lumen due to sample preparation artefact. (*b*) DAPI and ß-III-tubulin staining showing cell nuclei and nerve structures of the putative superficial neuromasts. (*c*) Autofluorescence image of *b*, showing variations in nuclei position, cell morphology and organization between cells forming the putative superficial neuromast and epidermis. (*d*) DAPI and ß-III-tubulin staining of a different possible superficial neuromast. (*e*) Autofluorescence image of *d* showing cell morphology and nuclei variations between cells forming the putative superficial neuromast and the epidermis. (*f*) Nerve crossing the dermis stained with DAPI and ß-III-tubulin. (*g*) Nerves crossing the dermis stained with Mallory trichrome. Canal neuromast, CN; dermis, D; epidermis, E; lateral line canal, LLC; nerve, N; potential superficial neuromast, SN?; unidentified tissue, UT. Scale bars in *b*–*g* = 50 µm.

Histologically, the continuous neuromast is composed of sensory hair cells, identified by their central location in the neuromast structure (sensory zone), by their nucleus facing the basal surface of the cell and by their organization in columns (figure 2*d* and figure 4*f*–*h*, electronic supplementary material, figure S4). Blood vessels and nerves are visible running immediately beneath the hair cells along the LLC (figure 4). An unidentified structure, composed of a group of cells, was found in the roof of the LLC (opposite to the neuromast; figure 4*f*–*h*). These cells stain with acid fuchsin (like epithelial cells) and did not test positive for β-III-tubulin, indicating the absence of nervous tissue in the structure. In some cases, this structure was absent or, because of sample processing, appeared separated from the epithelium in some of the histology sections (e.g. figure 5*a*). Ciliary bundles of the hair cells and the cupula were not visible in any of our samples, most likely due to their loss during sample fixation.

Immunofluorescence revealed sensory structures positive for β-III-tubulin embedded in the epidermis covering the tail (figure 5). These structures were uncommon and observed only along the lateral sides of the tail, covering the areas devoid of pores (which, in contrast, were located at the dorsal and ventral sides; figure 5*a*). These structures were not located in pits and had a different cell morphology from the surrounding epidermal cells and we identify them as putative superficial neuromasts (figure 5*b*–*e*). Exclusively in this region, nerves bordered the collagenous dermis surrounding the LLC and ran toward the epidermis (figure 5*f*–g).

## 4. Discussion

**Figure 6.**
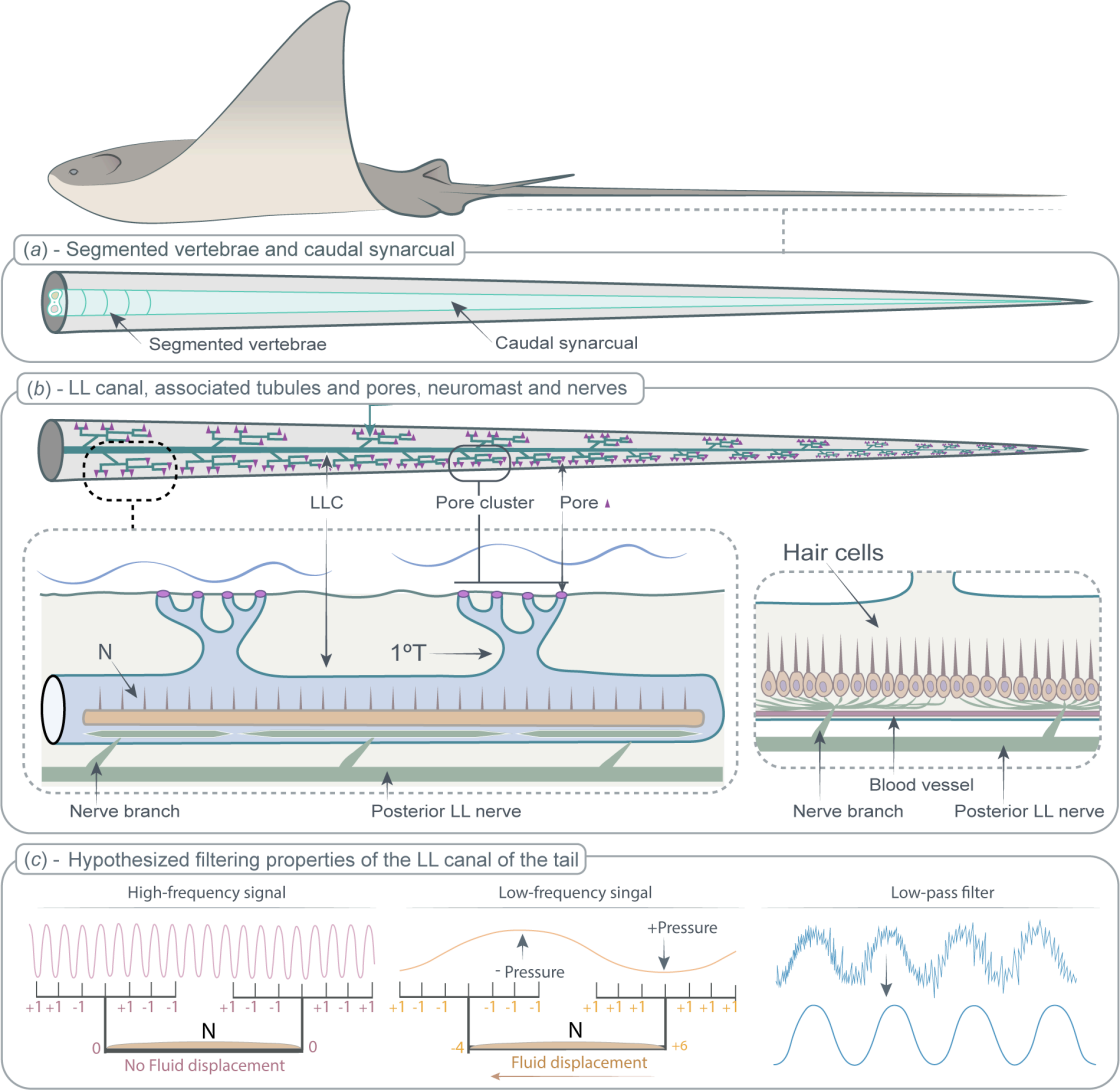
Anatomy and hypothesized function of the tail of *Rhinoptera bonasus*. (***a***) Vertebral morphology variation along the tail. At the base (proximal to the body), the vertebral column is formed by separated and well-defined vertebral units that allow active control of the tail. Caudally, vertebrae are fused, forming the caudal synarcual, which provides passive stiffness to the tail. (*b*) The lateral line canal (LLC), with branched tubules that open to the surrounding water via pores. The LLC contains the continuous neuromast. The continuous neuromast is connected via nerve branches to the posterior lateral line nerves (PLLNs). (*c*) The hypothesized filtering properties of the LLC in the tail suggest that the spatial distribution of the pores function as a low-pass filter, allowing lower frequency stimuli to pass while filtering out higher frequency ones. Additionally, the small pore diameter could filter out lower pressure (lower amplitude) stimuli, as higher pressure (i.e. higher amplitude) stimuli are required to induce fluid movement within the LLC. Lateral line canal, LLC; primary tubule, 1°T; continuous neuromast, N; posterior lateral line nerve, PLLN.

Using a combination of different imaging techniques, this study describes that the tail of *Rhinoptera bonasus*, the cownose ray, is a long and slender structure with a complex internal anatomy. The tail is characterized by having a vertebral column with an unusual morphology and by the presence, at both sides of the tail, of an LLC connected to a highly branched network of tubules that contains what appears to be a continuous neuromast. Preliminary analyses of tail morphology in other myliobatid species confirms that these features are not unique to cownose rays. In figure 6, we present a graphical summary of the main morphological results and further interpretation and hypothesis of the lateral line stimulus filtering capacity.

### (a) Vertebral anatomy and caudal synarcual

The tail of *R. bonasus* is an extension of the vertebral column that shows significant variation along the tail. At the base (between the pelvic fins and the posterior end of the barb), the vertebral column is formed by sequentially interconnected vertebrae (like other parts of the body, [20]) attached to a substantial musculature. However, from the posterior end of the barb to the tip, the muscular content is reduced, and the vertebral column is reshaped into a fused mineralized rod, forming a continuous mineralized structure not divided by vertebral segments (figure 6*a*). This fused structure is similar to the synarcual described in batoid fishes [21,22].

In chondrichthyans (especially chimaeras and batoids), the synarcual is defined as that portion of the vertebral column with fused segments (similar to the sacrum and pygostyle in tetrapods; [21–24]). In batoids, the synarcual within the body is a synapomorphy (i.e. shared derived feature of this clade; [25]). It is located in the anterior part of the vertebral column connected to the chondrocranium and expanding beyond the pectoral fins [21,26]. Due to its plate-like morphology, the synarcual has a biomechanical function, as it provides support to the enlarged pectoral fins [3,22,27]. Myliobatiforms are the only batoid group that has a second thoracolumbar synarcual located in the posterior area of the body (posterior to the pectoral fins), although its function is not known [25,28]. The fused caudal vertebrae structure of cownose rays’ tail is similar in several ways to the synarcual described in batoids, including being a continuous fused skeletal structure covered by tesserae, lacking vertebral centra, and containing a space for the spinal cord and the haemal canals that transverse the mineralized tissue through foramina. Therefore, we propose the term ‘caudal synarcual’ for the fused vertebrae structure of the *R. bonasus’* tail.

Variation in skeletal morphology along the cownose’s tail suggests variation in tail flexibility and mobility. The articulated vertebrae in the base zone of the tail, combined with the larger mass of tail musculature located here, likely allow overall tail mobility, support (the tail is most often held horizontally behind swimming rays, and does not sag ventrally) and orientation of the entire tail structure. Further, the caudal synarcual increases passive stiffness in the middle and distal tail regions and, in combination with the reduced muscle mass, should limit mobility and allow little deformation of the distal half of the tail. Therefore, our anatomical data suggest that cownose rays could control overall tail orientation from the base, while the more distal stiff tail region would be relatively incapable of independent bending or undulation (figure 6*a*). This result suggests that the tail is not an active propulsive structure contributing to thrust generation during locomotion, which contrasts with the slender tails in other vertebrates, such as the tapered tails of lizards or the caudal region of bony fishes which are articulated and mobile along their length [11,29]. The caudal synarcual of the *R. bonasus* tail may be important for increasing tail stiffness that will enable the tail to remain elevated to avoid contact with the substrate during foraging, to assist in maintaining stability while swimming and importantly, as a stable platform for the remarkable lateral line sensory system located within the tail.

The tails displayed dark coloration on the dorsal side, resulting from a high concentration of melanophores (melanin-producing chromatophores [30]). These melanophores were in the dermis (directly beneath the epidermis) as well as within the epidermis itself. While the presence of melanophores in the dermis is commonly reported in fishes, including batoids [31,32], their occurrence in the epidermis is rare and has not been documented in other elasmobranchs.

### (b) The mechanosensory lateral line

In bony fishes, the lateral line system (LLS; comprising the LLC, tubules, and both canal neuromasts and superficial neuromasts) is mechanosensory, as it detects water movements that cause pressure gradients among pores, moving the fluid that fills the tubules and canals [33,34]. This fluid movement causes a displacement of the cupula that covers the neuromasts, producing a deflection of the kinocilia and stereocilia of the hair cells and, ultimately, informing the central nervous system of the stimuli [33,35,36]. The presence of the LLC with neuromasts indicates that the tail of *R. bonasus* is a mechanosensory structure, able to detect hydrodynamic stimuli generated by movements of the surrounding water (such as movements generated by nearby rays, a predator, prey items or the body of the ray itself) (figure 6*b*).

Structurally, the LLC of the tail of *R. bonasus* contrasts with the posterior LLC (found in the trunk and tail regions) described in sharks and teleost fishes [37,38]. The LLC in the tail of *R. bonasus* opens to the dorsal and ventral surface of the tail via branched tubules (figure 6*b*). This result differs from sharks and teleosts [37,39], in which the LLC opens to the lateral sides of the trunk and tail through short, unbranched tubules (but see [40]). However, the LLC connected to a branched tubule network has been described in the body of other stingrays [6,7,38], which may be indicative of the importance of branching tubules in the biology of other myliobatiforms. In *R. bonasus*, the lateral line tubules and canals are surrounded by a collagenous tissue that varies in thickness proportional to canal diameter. Similar to the tunica media in the circulatory system [41], variation in thickness may indicate a mechanical role in maintaining the shape of the canals and tubules. Our results also show a group of cells located opposite to the neuromast in the LLC, which have not been previously described in other LLC. It was not possible to determine whether these cells form a continuous or discrete structure within the canal and, while the precise function of these cells remains unclear, our results indicate that they do not contain nervous tissue.

In the natural environment, hydrodynamic stimuli produced by biotic and abiotic sources are highly variable and include surface waves, local currents, prey, predators, conspecifics and body deformations [42,43]. Some of these stimuli could be considered environmental noise (unwanted water movements that interfere with the reception of a relevant stimulus) and the structure of the tail LLC and tubules can filter out these unwanted stimuli, enhancing the detectability of relevant stimuli at particular frequencies and amplitudes [33,44,45]. Filtering properties are determined by the structure of the neuromast cupula as well as the morphology of the LLC and tubules [46,47]. We suggest that differences in branching pattern, cross-sectional area, pore distribution and pore density determine the sensitivity and filtering properties of the LLS in the tail of *R. bonasus* (figure 6*c*; [7,44–47]).

Based on Laplace’s law and simulations correlating lateral line morphology and filtering properties [44,45], the reduction of cross-sectional area from the LLC to pores indicates that high pressures are required at the pore surface to move the fluid within the LLC (resulting in a deflection of the cupula and, thus, the hair cells of the neuromasts). In the tail of *R. bonasus,* the diameter is reduced from the LLC to tubules to pores and, therefore, the effective Reynolds number will be reduced, which reflects the corresponding increase in viscosity of the fluid and also acts to filter external signals. Since the pressure of a wave is correlated with its amplitude (higher amplitude waves produce higher pressures), this suggests that the LLS of the tail may function as a high-amplitude pressure detector (where low-amplitude waves would not produce movement of the fluid in the LLC). Non-pored canals, which are thought to have a tactile function in other batoids [7,48], are absent in the tail of *R. bonasus.*

The sensitivity of LLSs is influenced by a combination of factors, including the tree-like topology of the network, as well as the density and spatial distribution of pores [7,44,45]. A highly branched network of tubules allows an increased number of pores to connect the neuromasts to the surrounding water. An increased number of pores expands the area capable of detecting hydrodynamic stimuli, allowing *R. bonasus* to perceive water movements along the entire length of its tail [44].

The distribution of the surface pores can determine which frequencies are being filtered out (i.e. not detected; [44,45]). Each pore cluster on the tail of *R. bonasus* contains around 15–20 pores arranged linearly and closely spaced. The pressure differences among pores within the same cluster should be averaged when reaching each primary tubule. When the wavelength of a given stimulus is similar to the distance between two pores, the pressure difference in the tubule bifurcation will be zero, resulting in no fluid displacement (figure 6*c*). Conversely, stimuli with lower frequencies will produce a pressure difference between two primary tubules, causing fluid displacement within the canal where the neuromast is located. The averaging of pressure differences in the tubules could also allow them to function as spatial filters, attenuating the transmission of higher frequency flows and emphasizing lower frequency stimuli. Thus, the LLS in the tail of *R. bonasus* could function as a low-pass filter (figure 6*c*).

No differences were observed in the pattern and distribution of pores between sexes. However, variations related to lateral line diameter, animal size and tail zone were observed. First, the tail LLS (including canal, tubules and pores) appears to scale with the size of the animal, although ontogenetic studies are needed to verify this result. For each tail zone along the length of the canal (base, middle and tip), the middle region of the tail contains the highest number of branching tubules and, consequently, the highest density of pores. This suggests that this area is more sensitive than the base and tip of the tail.

### (c) The continuous neuromast

We have demonstrated that the neuromast within the LLC has a *continuous* structure along the entire length of the tail (figure 6*b*). This continuous neuromast appears to be composed of a continuous epithelium with numerous hair cells, as no breaks in the epithelium were observed during the analysis of any of our samples. However, due to the difficulty of imaging the gelatinous cupula (if present), it is not known whether the cupula also has a continuous structure. This continuous neuromast differs from what is known in teleost fishes, where neuromasts are discrete structures located between the division of primary tubules that extend to the skin surface [36,39]. Long neuromasts have been diagrammed once previously in elasmobranchs, although direct demonstration of the continuity of neuromasts has not been previously shown [2,6,49], especially over such an extensive distance as described in the tail of *R. bonasus*. In contrast, discrete neuromasts have been reported in the LLC of the head in embryos of the little skate (*Leucoraja erinacea*; [50]). The difference in neuromast length (continuous versus discrete) between *R. bonasus* and *L. erinacea* may be related to three possible options: (i) neuromasts may grow in length with ontogeny (as described in bony fishes [51,52]), forming what appears to be a continuous structure in adult batoids, (ii) neuromast length may vary across different body regions in both rays and skates, or (iii) neuromast length may differ among batoid species. Further research is needed to explore these hypotheses.

The cross-sectional anatomy of the neuromasts in *R. bonasus* shows that the neuromast is similar in morphology and position within the canal to those in bony fishes [39,51]. Branches of the PLLNs innervate the continuous neuromast periodically, between the position of two primary tubules. This confirms the hypothesis proposed by [49] where, although the neuromast appears to have a continuous epithelium, hair cells are divided into packages or groups, with the information collected by these cell groups being transported by a specific nerve branch (figure 6*b*). This division would allow the information to be localized spatially, so the animal may be able to recognize where along the LLC a hydrodynamic disturbance may be located. Further studies are needed to understand the function and advantages given by continuous neuromasts and to determine whether this is a conserved structure across other species of chondrichthyans.

We also observed other sensory structures in the epidermis covering the tail. These structures were uncommon and only visualized using immunofluorescence. Although the cupula was not preserved, we believe that these structures are superficial neuromasts. Superficial neuromasts have been described in the tails of myliobatiforms such as *Dasyatis sabina* [6] and *Myliobatis australis* [53] located in pits (‘pit organs’). However, the putative superficial neuromasts found in this study do not occur in pits but are embedded in the epidermis and are not associated with the small tail denticles (identified as claw type prickles, [54]). These putative superficial neuromasts in the cownose ray tail were scarce and distributed on the skin along both lateral sides of the tail, areas devoid of lateral line pores. This agrees with the distribution of superficial neuromasts previously described in other batoid tails [6,55].

## 5. Conclusion

Myliobatid stingrays are characterized by having a long and slender tail that extends behind the body. A tail with this morphology is characteristic of the group and is not used to generate thrust during locomotion. We used a combination of imaging techniques to describe the anatomy of the tail in *Rhinoptera bonasus* (the cownose ray) and our results show that the tail is stiffened due to the presence of the caudal synarcual. This likely increases tail stability by passively damping tail perturbations during swimming as well as reducing hydrodynamic noise by limiting tail movement. The tail also contains a component of the posterior lateral line system, comprised of a bilateral pair of lateral line canals on each side of the tail, each of which is connected to an extensive tubule network and contains what appears to be a continuous neuromast. This branched tubule network is proposed to act as a filter, blocking signals with higher frequencies and lower amplitudes, ultimately influencing the hydrodynamic stimulus received by the neuromasts. Therefore, we hypothesize that this network acts as a low-pass filter. Cownose rays are active swimmers living in a diversity of environments where background noise can be high; for example, noise produced by waves near the surface or during swimming (signals associated with high frequencies). The filtering capacities of the lateral line system of the tail could improve the signal-to-noise ratio, avoiding overstimulation of the canal neuromasts. We propose that the tail of *R. bonasus* may act as a hydrodynamic antenna able to detect water disturbances resulting from prey, predators, body movements and near body flow dynamics.

## Data Availability

Raw data from micro-computed tomography, confocal microscopy, fluorescence microscopy and light microscopy are available [56]. Supplementary material is available online [57].
